# Supplementary material for: Splice-Junction-Based Mapping of Alternative Isoforms in the Human Proteome
Source: Cell Rep. Author manuscript; Available in PMC 2020 Jan 15. (PMC6961840; doi:10.1016/j.celrep.2019.11.026)
Supplement: 2 [file NIHMS1546469-supplement-2.zip › DF1/PXD010154/Liver/Liver_39_CYB5R1_GPQANISTSPPELMAAWSSGHTLLSPVMR.pdf]

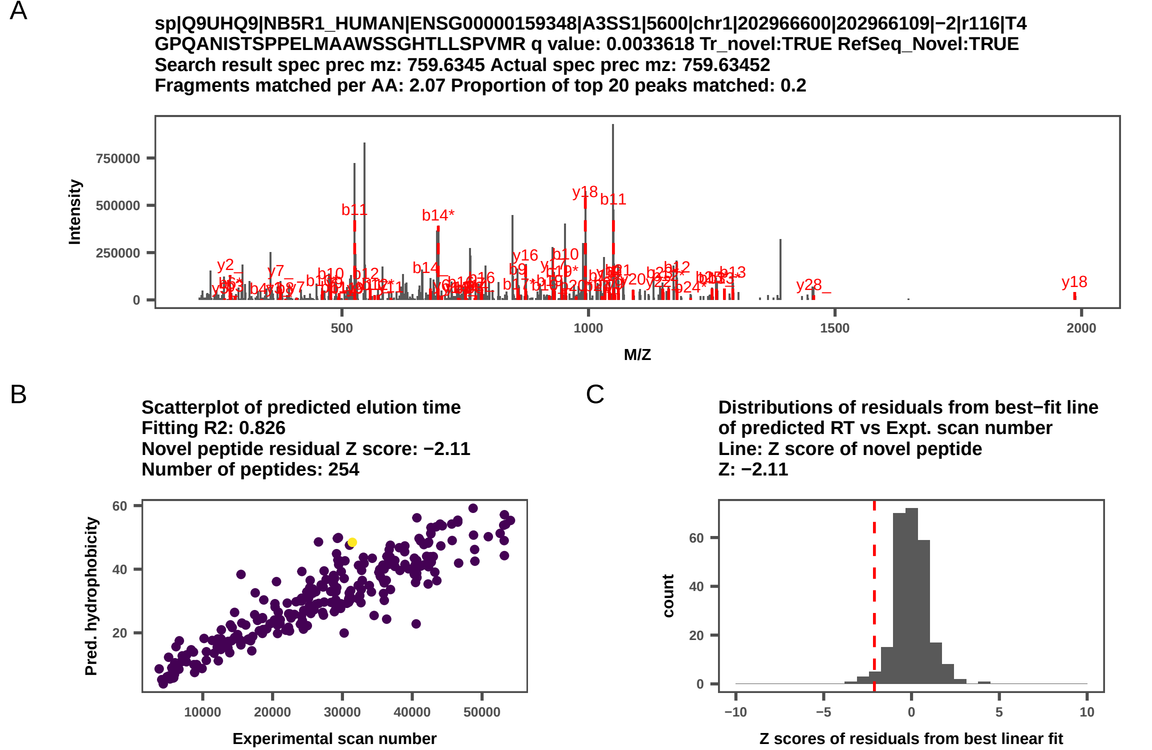
A
sp|Q9UHQ9|NB5R1_HUMAN|ENSG00000159348|A3SS1|5600|chr1|202966600|202966109|−2|r116|T4
GPQANISTSPPELMAAWSSGHTLLSPVMR q value: 0.0033618 Tr_novel:TRUE RefSeq_Novel:TRUE
Search result spec prec mz: 759.6345 Actual spec prec mz: 759.63452
Fragments matched per AA: 2.07 Proportion of top 20 peaks matched: 0.2
Intensity
750000
500000
250000
0
b11
b14*
y18
b11
y16
b10
b9
y2_
y7_
b10
b12
b14_
b16
b12
b13
y28_
y18
500
1000
1500
2000
M/Z
B
Scatterplot of predicted elution time
Fitting R2: 0.826
Novel peptide residual Z score: −2.11
Number of peptides: 254
Pred. hydrophobicity
60
40
20
10000
20000
30000
40000
50000
Experimental scan number
C
Distributions of residuals from best−fit line
of predicted RT vs Expt. scan number
Line: Z score of novel peptide
Z: −2.11
count
60
40
20
0
−10
−5
0
5
10
Z scores of residuals from best linear fit
